# Establishing Expert Consensus on Competencies for Safe Ward‐Based Postoperative Epidural Analgesia Management: Protocol for a Modified Delphi Study

**DOI:** 10.1111/aas.70275

**Published:** 2026-06-16

**Authors:** Cornelia Charlotte Lamprecht, Søs Bohart, Kim Wildgaard, Morten Vester‐Andersen, Anne Mørup‐Petersen, Thordis Thomsen

**Affiliations:** ^1^ Department of Orthopaedic Surgery Copenhagen University Hospital, Herlev‐Gentofte Herlev Denmark; ^2^ Herlev Anaesthesia Critical and Emergency Care Science Unit (ACES), department of Anaesthesiology Copenhagen University Hospital, Herlev‐Gentofte Herlev Denmark; ^3^ Department of Clinical Medicine University of Copenhagen Copenhagen Denmark; ^4^ Department of Orthopaedic Surgery Copenhagen University Hospital, Bispebjerg Copenhagen Denmark

**Keywords:** clinical competence, Delphi technique, epidural analgesia, nursing education, perioperative care, postoperative pain management

## Abstract

Postoperative epidural analgesia (EA) is an important analgesic strategy for selected surgical procedures. However, clinical practice and training vary across settings, and no consensus‐based framework currently defines the competencies required for safe ward‐based EA management. A modified Delphi study will establish expert consensus on competencies for ward‐based postoperative EA management. Items derived from prior empirical work will be rated by experts across three Delphi rounds: two rounds in which experts rate the relevance of each item on a seven‐point Likert scale with controlled feedback between rounds, followed by a third round in which experts rate the required competence level for included items using Miller's pyramid. The study is expected to generate consensus on essential competencies and their required level for safe ward‐based postoperative EA management. The findings will inform the development of a competency‐based training framework and may contribute to more consistent practice and improved patient safety.

## 1 Introduction

Postoperative epidural analgesia (EA) is an important analgesic strategy for selected surgical procedures and patient groups, requiring timely assessment, recognition of side effects, and safe management by the clinical team. In surgical ward settings, this responsibility falls primarily to nursing staff, who provide continuous bedside care throughout the postoperative period [1, 2, 3, 4]. Despite the importance of EA, international research highlights significant variation in monitoring practices, escalation routines, clinical decision‐making, and access to structured education [4, 5, 6, 7]. Such variability compromises consistency of care and poses potential patient safety risks.

International guidelines address safety recommendations for postoperative EA management, but vary in scope and operational detail, with limited guidance on ward‐based nursing practice and required competencies [2, 8, 9]. In particular, existing educational focus tends to address the technical placement and initiation of EA, with comparatively less attention to ongoing ward‐based management.

National Danish data reflect these challenges: A recent nationwide survey found considerable variation in ward nurses' management of breakthrough pain during EA, with organisational barriers, inconsistent access to formalised education and competence gaps reported across surgical wards [10]. Our scoping review similarly identified heterogeneity in the content, structure and evaluation of educational interventions for EA management [4]. A qualitative multicentre focus group study further revealed variation in knowledge, clinical reasoning and organisational support among post‐anaesthesia care unit (PACU) and surgical ward nurses, with an absence of clear responsibility allocation (unpublished data). Together, these findings underscore the need for a unified framework for safe and consistent ward‐based EA practice.

## 2 Objectives

This Delphi study aims to establish expert consensus on the core competencies to be included in a structured training programme for the safe management of postoperative EA in surgical ward settings.

Specifically, the study seeks to achieve consensus on:

- Essential competencies required for safe and effective postoperative ward‐based EA management.
- The required level of each competency

The resulting consensus will inform the development of a competency‐based training framework for postoperative EA management for surgical ward nurses.

## 3 Methods

### 3.1 Reporting Standards

This protocol follows the DELPHISTAR reporting guidance [11], and is informed by established methodological recommendations for Delphi studies [12, 13, 14]. Specifically, the five key recommendations outlined by Humphrey‐Murto et al. [12] concerning method selection, questionnaire development, participant identification, consensus threshold definition, and justification of methodological modifications are addressed throughout this protocol and are discussed in the Discussion section.

### 3.2 Study Design

**FIGURE 1:**
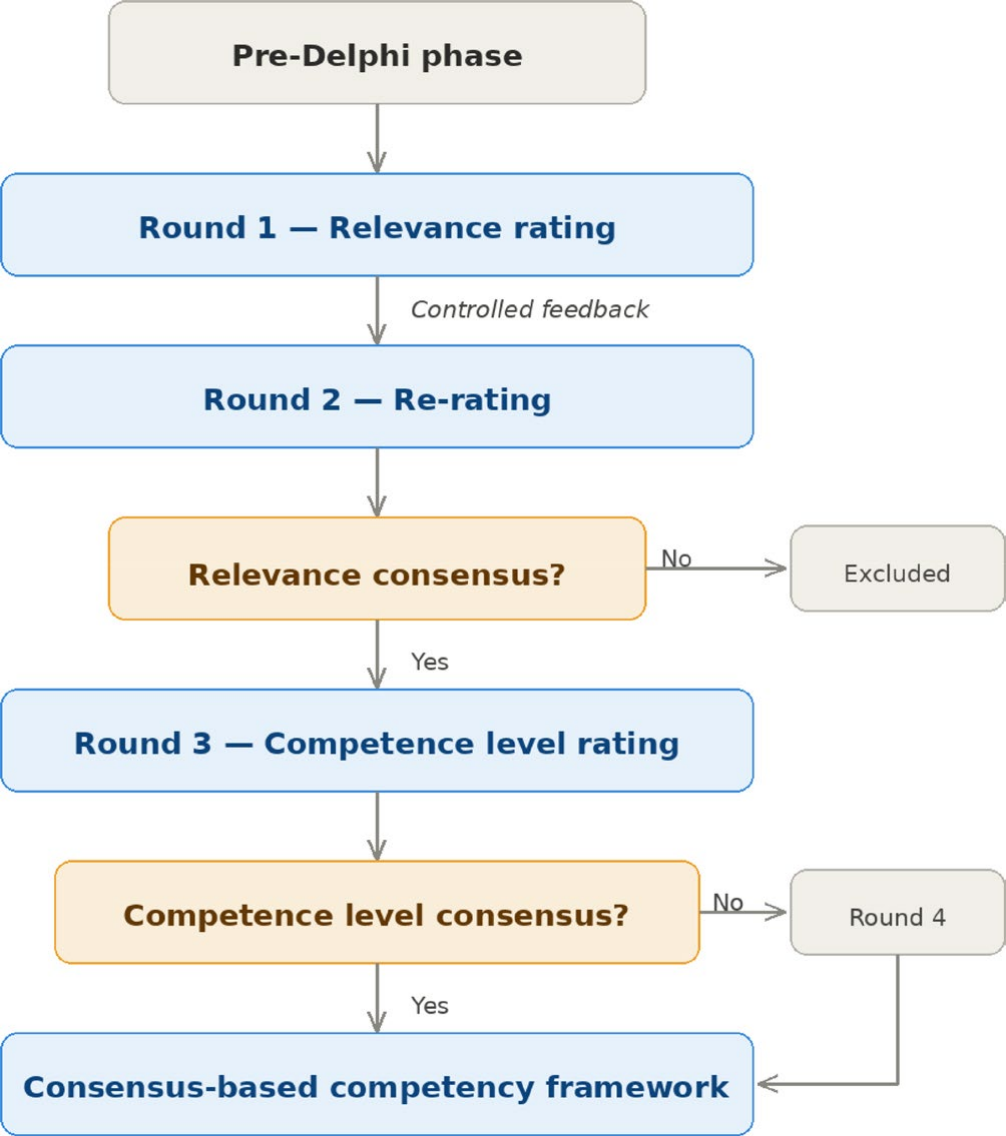
Overview of the modified Delphi study process.

The study will use a modified Delphi design [12, 15]. The process will include a preparatory pre‐Delphi phase followed by three iterative Delphi rounds. Rounds 1 and 2 focus on establishing consensus on the relevance of each item using a Likert scale with controlled feedback between rounds. Round 3 addresses the required competence level for items that have reached consensus on relevance, using Miller's pyramid [16]. A fourth round will be conducted if consensus is not achieved in Round 3 (Figure 1).

Participant anonymity will be maintained throughout the Delphi process to minimise dominance bias and group conformity [12, 13, 14].

### 3.3 Participant Eligibility

In this study, ‘experts’ are defined as healthcare professionals with substantial clinical experience in postoperative EA management and/or responsibility for EA‐related education in perioperative settings. Expertise is operationalised through professional role, years of clinical experience, and domain‐specific knowledge, consistent with established Delphi guidance [13, 17].

Eligible participants must meet at least one of the following criteria:

- Registered nurses working in PACUs or surgical wards with substantial clinical experience and regular involvement in the management of postoperative EA.
- Consultant anaesthesiologists (board‐certified specialists) with clinical experience in postoperative pain management and EA.

Substantial clinical experience will be interpreted in relation to both duration and depth of involvement in EA management, rather than a predefined minimum number of years. Priority will be given to participants with additional roles in education, training, or clinical leadership, such as responsibility for staff training, involvement in guideline implementation, or key roles in acute pain management teams.

Healthcare professionals unable to commit to participation in all Delphi rounds will be excluded.

### 3.4 Sampling and Panel Composition

A purposive sampling strategy will be used. Participants will be recruited from the orthopaedic and abdominal surgical departments that use postoperative EA and participated in a Danish national survey on postoperative EA management in 2023 [10], response rate 84%. All 61 departments represented in the survey, along with their associated anaesthesiology departments (including PACUs), will be contacted. This approach supports targeted identification of clinical environments with established experience and interest in EA care while supporting national representation.

To ensure variation in perspectives, the sample will include participants across professional groups (surgical ward nurses, PACU nurses, and anaesthesiologists), clinical settings (surgical wards and PACUs), and hospital types (e.g., university and regional hospitals). Chief nurses or nurse managers from surgical wards and PACUs, as well as heads of anaesthesiology departments, will be invited to nominate clinicians with relevant clinical expertise. Departments will be asked to nominate 1–2 nurses with current clinical experience in EA management and one anaesthesiologist with relevant expertise [12].

All nominated individuals will be screened against predefined eligibility criteria prior to inclusion. To ensure adequate representation of PACU nursing perspectives, particular attention will be given to the inclusion of PACU nurses alongside surgical ward nurses in the recruitment process.

A minimum of 30 participants is considered sufficient for a stable and information‐rich Delphi panel [14]. However, a larger panel is anticipated given the breadth of the recruitment strategy. Panel size will not be artificially restricted, as broader representation across professional groups, clinical settings, and hospital types is expected to strengthen the robustness and transferability of the consensus findings. Invitations will be distributed by email with two reminders at one‐week intervals.

Participants who do not complete Round 1 will not be invited to subsequent rounds. Attrition across rounds will be monitored and reported.

### 3.5 Delphi Procedure

Table 1 provides an overview of the data collected and outputs generated across all phases of the Delphi process.

**TABLE 1:** Data collection, participants, and outputs across Delphi rounds.

Phase/Round	Participants	Objective	Data collected	Output
Pre‐Delphi	Project group	Item development and refinement	Structured evidence extraction and item refinement log	Predefined item list organised into content domains
Delphi Round 1	Full expert panel	Initial rating of item relevance using 7‐point Likert scale	Relevance ratings (1–7 scale) and free‐text comments	Preliminary ratings (median scores, proportions in the upper range, response distributions)
Delphi Round 2	Full expert panel	Re‐rating with controlled feedback	Re‐ratings and free‐text comments	Consensus‐based item list (Items meeting relevance consensus criteria)
Delphi Round 3	Full expert panel	Rating of required competence level for items that reached relevance consensus	Competence level ratings using Miller's pyramid [1, 2, 3, 4] and free‐text comments	Consensus‐based competency framework with required competence levels
Delphi Round 4 (if required)	Full expert panel	Re‐rating of items not reaching competence level consensus in Round 3	Re‐ratings	Final consensus decisions

### 3.6 Pre‐Delphi Preparation and Item Development

Prior to initiating the Delphi rounds, a structured preparatory pre‐Delphi phase will be conducted to ensure content validity and clinical relevance of the initial item pool. This phase encompasses item generation, domain organisation, project group review, and pilot testing of the questionnaire.

### 3.7 Item Generation, Evidence Synthesis and Domain Organisation

The initial pool of Delphi items will be generated through synthesis of four complementary evidence sources:

1. A scoping review mapping existing educational interventions for EA management [4]
2. A qualitative focus group study exploring nurses' experiences of postoperative EA management (unpublished data)
3. Findings from a national Danish survey on ward nurses' management of EA [10]
4. Relevant international clinical guidelines and consensus statements addressing ward‐based EA management [2, 8, 9]

Items will be organised into preliminary content domains to ensure comprehensive coverage of relevant competencies. In the Delphi questionnaire, items will be presented within their respective domains to support respondent orientation and contextual understanding. Within each domain, items will be presented in a fixed order. Participants will be instructed to evaluate each item independently, without regard to its domain classification. This approach balances navigability with independent item evaluation. The final structuring of competencies will be undertaken by the research team, informed by the Delphi consensus results.

### 3.8 Project Group (Preparatory Phase)

A project group (*n* = 6), comprising three nurses and three physicians with clinical and research expertise across anaesthesia, orthopaedic surgery, and Delphi methodology, will contribute to the development and refinement of the initial item pool.

The group will review the structured evidence synthesis, refine item wording, structure content domains, and identify potential overlaps or ambiguities. The group will not prioritise, rate, or exclude items or have any influence on the consensus process. Members of the project group will not participate in the Delphi rounds.

### 3.9 Pilot Testing of Delphi Questionnaire

A pilot phase will be conducted prior to Round 1 to assess clarity, relevance, and feasibility of the questionnaire. This will include cognitive pretesting with a small sample of healthcare professionals (*n* = 3–5) representing the target groups, and a technical pilot test (*n* = 3–5) using the full survey in REDCap to evaluate functionality, completion time, and usability.

Pilot participants will be selected to reflect the intended expert panel and will be excluded from the final Delphi panel where feasible. Minor revisions to item wording, structure, and response options will be made as needed, without altering the underlying conceptual content.

### 3.10 Round 1—First Consensus Rating

In Round 1, participants will rate the relevance of each predefined item for inclusion in a training programme for ward‐based EA management using a 7‐point Likert scale (1 = not at all relevant, 7 = highly relevant). Participants will be instructed to rate each item based on what they consider important for safe and effective practice, rather than based on current practice or local feasibility.

An ‘Unable to rate/Outside my expertise’ option will be available for all items. Participants will also be invited to propose new items through an open free‐text field at the end of the questionnaire. New items proposed by participants will be reviewed by the project group and, if deemed within scope, included in Round 2, consistent with established Delphi practice [13, 14].

### 3.11 Round 2—Re‐Rating With Controlled Feedback

In Round 2, participants will be provided with controlled feedback from Round 1, including median scores and the distribution of ratings for each item. Provision of controlled feedback between rounds is a defining feature of the Delphi method, allowing participants to reconsider their ratings in light of group responses while maintaining independent judgement [13, 14].

Participants will be invited to reconsider and re‐rate all items using the same rating scale as in Round 1. The same instructions will apply, including rating items based on what they consider relevant for safe and effective clinical practice, and the option to indicate ‘Unable to rate/Outside my expertise’.

### 3.12 Round 3—Competence Level Rating

Items reaching relevance consensus after Round 2 will be included in Round 3. Participants will be asked to rate the required competence level for each included item using Miller's pyramid of clinical competence [16], which ranges from theoretical knowledge (1 = Knows) to independent clinical performance (4 = Does). Participants will be instructed to rate the level that a surgical ward nurse should ideally achieve upon completing a training programme in postoperative EA management.

Controlled feedback from Round 2 (item inclusion list with relevance consensus data) will be provided to contextualise the rating task. An ‘Unable to rate/Outside my expertise’ option will be available for participants to elaborate on their competence level ratings where desired.

### 3.13 Consensus Criteria

Consensus criteria are defined separately for relevance (Rounds 1–2) and required competence level (Round 3).

Relevance consensus (Rounds 1–2) will be considered achieved when the median score is ≥ 6 and ≥ 70% of participants rate the item within the upper range (scores 6–7). A median threshold of ≥ 6 on a 7‐point scale was selected to ensure that only items with strong and broad expert endorsement are retained, consistent with established Delphi practice [13, 14]. Items meeting the relevance consensus criteria after Round 2 will proceed to Round 3 for competence level rating. Items not meeting relevance consensus will be excluded from the core framework but may be reported as areas of non‐consensus. Competence level consensus (Round 3) will be defined as ≥ 70% of participants rating the item at the same level (or within one adjacent level) on Miller's pyramid. Items not reaching competence level consensus in Round 3 will be subject to a fourth round if needed.

### 3.14 Data Analysis

Quantitative data will be analysed using descriptive statistics. For each item and round, median scores, proportions of ratings in the upper range (scores 6–7), and response distributions will be calculated. Median scores and percentage agreement are used as the primary measures of central tendency and consensus, as these are appropriate for ordinal data and are consistent with established Delphi practice [13, 14]. Stability across rounds will be assessed by comparing these measures between rounds.

Participant characteristics will be reported descriptively, including professional role, years of clinical experience in EA management, clinical setting, and hospital type, to allow assessment of panel composition and representativeness.

Qualitative comments from Round 1, including proposed new items, will be reviewed to identify ambiguities, clarify item wording, and support interpretation of quantitative findings, prior to Round 2. New items deemed within scope will be included in Round 2. Comments will be summarised narratively to describe common rationales and suggested refinements. Qualitative data will not be used to modify the underlying conceptual content of items but may inform clarification of item wording prior to Round 2.

### 3.15 Data Management

Survey data will be collected and stored in REDCap on secure institutional servers compliant with GDPR [18, 19]. Participants will be assigned unique study IDs, and all data will be de‐identified prior to analysis.

### 3.16 Patient and Public Involvement

Patients and the public are not involved in this study, as it focuses on professional expertise related to clinical competence and educational design. No patient data will be collected. The final framework is expected to indirectly benefit postoperative patients through strengthened ward‐based EA safety and practice consistency.

### 3.17 Ethical Considerations

The study will be conducted in accordance with the principles of the Declaration of Helsinki and applicable European data protection regulations.

The study has been registered and approved in the Capital Region of Denmark's data protection system, Privacy (registration number: p‐2026‐20,629), and is registered in OSF Registries (registration number: Kmws8, https://osf.io/Kmws8). Electronic informed consent will be obtained from all participants. The Ethics Committee in the Capital Region was notified (F‐26003352) but waived full ethical approval in accordance with Danish legislation (waiver‐date: 28.02.2026).

## 4 Discussion

This protocol describes a modified Delphi study designed to establish a consensus‐based competency framework for safe and effective ward‐based management of postoperative EA. Delphi methodology is well suited for developing consensus on complex clinical and educational questions, particularly when empirical evidence is limited, or practice varies [12]. By integrating findings from prior empirical work with perspectives from anaesthesiologists, PACU nurses, and surgical ward nurses, the study aims to develop a clinically relevant and practice‐oriented framework.

A key strength of the study is the interdisciplinary composition of the expert panel, reflecting the collaborative nature of postoperative EA management. Inclusion of participants across professional groups and clinical settings is expected to enhance the relevance and applicability of the resulting framework to real‐world practice.

As with all Delphi studies, the findings will reflect expert consensus rather than empirical testing. Potential limitations include attrition between rounds and sampling bias related to recruitment from departments which previously participated in our national survey. In addition, the national context may influence transferability to other healthcare systems.

Several methodological measures have been applied to strengthen rigour and minimise bias. Participant anonymity is maintained throughout all Delphi rounds to reduce dominance bias and group conformity [13, 14]. Controlled feedback between rounds supports independent judgement while allowing participants to reconsider their responses in light of group trends, a defining feature of the Delphi method that distinguishes it from simple surveys [12, 14]. Predefined consensus criteria and the separation of item development from consensus rating further enhance transparency and methodological rigour.

The three‐round design and the separation of relevance from competence level assessment represent key methodological modifications to the standard Delphi design, made in accordance with the recommendations of Humphrey‐Murto et al. [12, 15]. Separating these two dimensions across rounds is methodologically important: it ensures that participants can revise their relevance ratings in response to controlled group feedback before the competence level dimension is introduced, supporting genuine iterative consensus‐building on each dimension independently. Miller's pyramid was selected for Round 3 because it accommodates the full range of item types—from theoretical knowledge (Levels 1–2) to independent clinical performance (Level 4, ‘Does’), which represents the expected ceiling for a surgical ward nurse.

## Author Contributions

**Cornelia Charlotte Lamprecht:** conceptualization, methodology, investigation, funding acquisition, visualization, writing – original draft, project administration. **Søs Bohart:** conceptualization, writing – review and editing. **Kim Wildgaard:** conceptualization, supervision, writing – review and editing. **Morten Vester‐Andersen:** conceptualization, supervision, writing – review and editing. **Anne Mørup‐Petersen:** conceptualization, supervision, writing – review and editing. **Thordis Thomsen:** conceptualization, supervision, writing – review and editing.

## Funding

This work was supported by Novo Nordisk Fonden (NNF25OC0106884).

## Disclosure

This study is registered in OSF Registries (registration number: kmws8, https://osf.io/kmws8).

## Conflicts of Interest

The authors declare no conflicts of interest.

## Data Availability

No datasets have yet been generated. Anonymised Delphi data will be made available on reasonable request following publication.
